# Bilateral conjunctival sporotrichosis in a domestic cat: case report

**DOI:** 10.3389/fvets.2025.1661507

**Published:** 2025-10-21

**Authors:** Joelson Cavalcanti Silva, Gabriella Menezes de Freitas Silva, Lucas Rannier Ribeiro Antonino Carvalho

**Affiliations:** ^1^Ophthalmology Department, SouVet Veterinary Hospital, João Pessoa, Brazil; ^2^Department of Physiology and Pharmacology, Karolinska Institutet, Stockholm, Sweden

**Keywords:** *Sporothrix* spp., veterinary ophthalmology, domestic cat, kittens, feline conjunctivitis

## Abstract

Feline sporotrichosis is a subcutaneous mycosis caused by the dimorphic fungi of the genus *Sporothrix*. It is a zoonotic disease that has been increasingly reported in Brazil. While it commonly presents as skin nodules, nasal discharge, and generalized ulcerative lesions, ocular involvement is rare. This case report describes an unusual presentation of sporotrichosis with bilateral conjunctival manifestations in a 2-month-old domestic mixed-breed kitten treated at a private veterinary hospital in João Pessoa. Clinical examination revealed hyperemic conjunctivae with a granulomatous appearance, follicles, chemosis, and mucopurulent secretion. Additional tests were requested, including complete blood count, tests for feline immunodeficiency virus (FIV) and feline leukemia virus (FeLV), antibiogram, fungal culture, and exfoliative cytology. The antibiogram identified *Enterococcus* sp., sensitive only to 10 μg gentamicin, and cytology revealed yeasts consistent with *Sporothrix* spp., which was subsequently confirmed by fungal culture. Treatment was adjusted with gentamicin eye drops, topical and oral itraconazole, hepatoprotector and a food supplement containing beta-glucans to aid healing. After 1 month of therapy, the lesions disappeared, leaving the only sequelae as adhesion of the third eyelid to the palpebral conjunctiva. Ocular sporotrichosis in kittens is a rare condition and, in this case, bilateral involvement was observed, which suggests the need to consider this disease in the differential diagnosis of cats with ophthalmic alterations, especially in urban environments with a high prevalence of the disease. Treatment began with oral itraconazole and topical adjuvant therapy, resulting in clinical remission after 4 months of treatment.

## Introduction

Species of *Sporothrix* are dimorphic, saprophytic, and cosmopolitan fungi found in soil and plant debris, particularly in temperate and humid tropical regions. The genus comprises several species, including *Sporothrix brasiliensis, Sporothrix globosa, Sporothrix mexicana,* and *Sporothrix luriei,* with *Sporothrix brasiliensis* being the most prevalent in Brazil (1, 2).

Sporotrichosis is a zoonosis that affects several species, including felines, humans, horses, dogs, pigs and cattle. Domestic cats play a significant role in the spread of the disease, especially intact male cats with access to the street. The skin lesions in these animals contain a large number of infective fungal cells, characterizing them as a notable source of infection (2, 3).

In Brazil, sporotrichosis is considered an emerging disease, with a higher incidence in regions with socioeconomic and environmental difficulties, which makes access to adequate treatment difficult. Treatment is often costly, and few city governments provide resources for managing the disease in animals (2, 4).

Clinically, sporotrichosis is a subcutaneous mycosis that may present in acute, subacute, or chronic forms. Lesions are classified as cutaneous, lymphocutaneous, disseminated, and extracutaneous, and may affect the lungs, joints, bones, and mucous membranes (5). In cats, multiple cutaneous lesions on the nasal region are common, characterized by nodules and ulcers that may progress to hemorrhagic crusts. Most cases present with three or more lesion sites, frequently located on the head, limb extremities, and the base of the tail (2, 6). While the cutaneous form is the most frequent, ocular involvement in cats, including conjunctivitis caused by *Sporothrix*, is uncommon and poorly documented, with only a few reports of granulomatous conjunctivitis associated with *S. brasiliensis* isolated from the palpebral conjunctiva (7). Considered an emerging disease, the infection may present atypically, hindering diagnosis and delaying the initiation of therapy. The condition may progress to severe complications, including zoonotic dissemination, drug resistance, and permanent ocular sequelae (2, 8, 9).

Diagnosis is based on the identification of *Sporothrix* spp., obtained through clinical examinations and the animal’s history. Additional tests include cytopathology of secretions and exudate aspirates, histopathology of lesions, serological tests, intradermal tests, inoculation in animals and polymerase chain reaction (PCR). Fungal culture is considered the gold standard for identifying *Sporothrix* spp. (10, 11).

The choice of therapeutic protocol for feline sporotrichosis considers the extent of the lesions, severity of the disease and general health of the patient, since immunosuppressed or weakened animals may present therapeutic failures. Itraconazole is the drug of choice for treatment, being more effective when compared to sodium or potassium iodides, previously used in both felines and humans (4, 6).

This report describes an atypical case of feline sporotrichosis with bilateral conjunctival involvement in a 2-month-old kitten, a presentation rarely documented in the literature. By reporting this unusual manifestation, we aim to alert veterinarians in endemic regions to consider sporotrichosis as a differential diagnosis for feline conjunctivitis, thereby facilitating earlier recognition and treatment.

## Case report

A male, mixed-breed kitten, approximately 2 months old and weighing 300 g, was referred to the ophthalmology service of a veterinary hospital in João Pessoa, PB, Brazil. The animal had been rescued from the streets and was recently placed in a household with no other animals. At the time of rescue, the kitten already presented ocular alterations that had been empirically treated as chronic bilateral conjunctivitis, with no response to conventional therapy. According to the caregiver, prior to the specialized consultation, the patient had received tobramycin and diclofenac eye drops, later combined with ciprofloxacin, ofloxacin, and chloramphenicol ophthalmic ointment, all used concomitantly until the initial evaluation.

**Figure 1:**
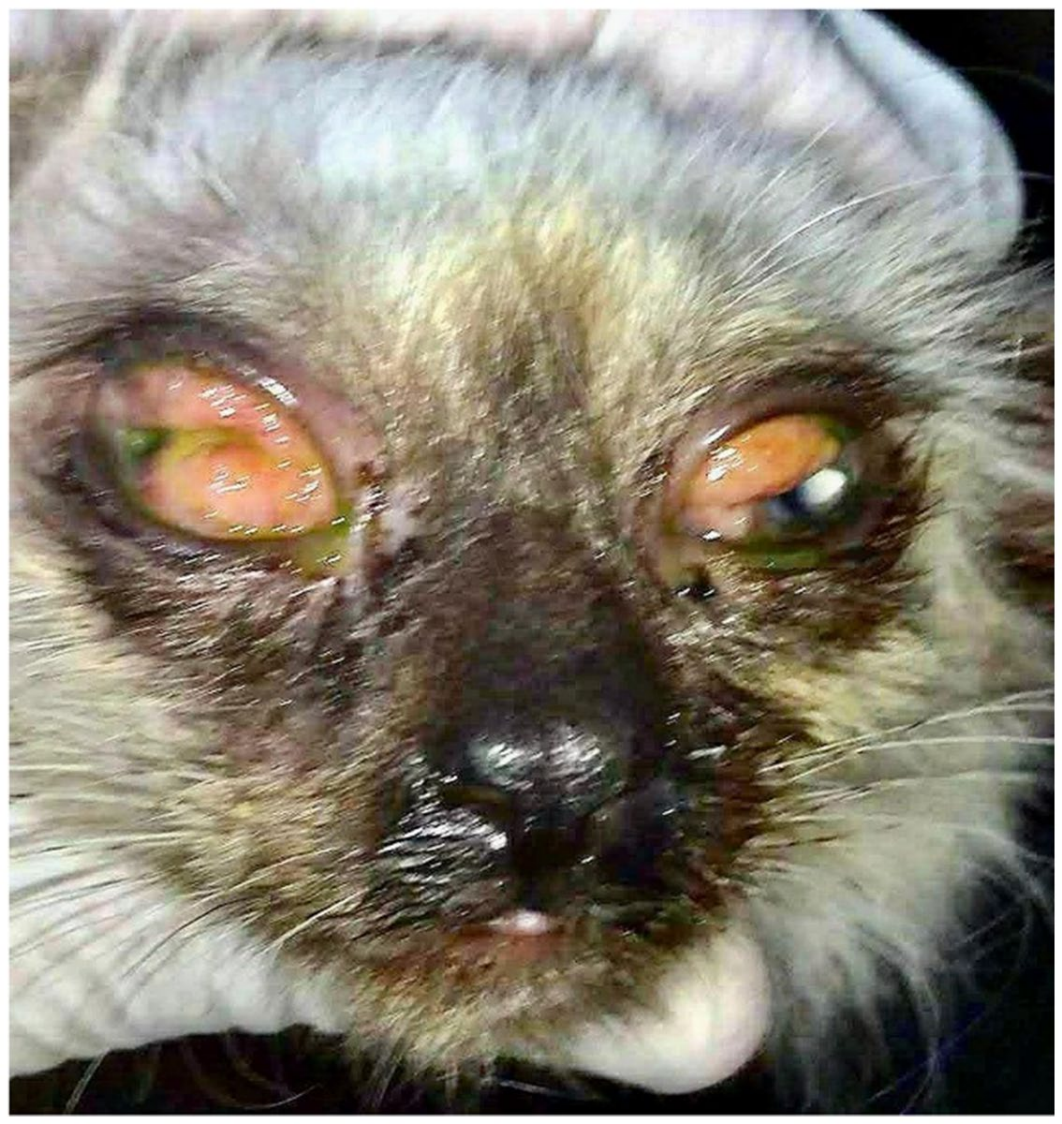
Initial presentation of the patient during ophthalmological evaluation revealed hyperemic conjunctiva with a granulomatous appearance, presence of follicles, chemosis, and mucopurulent discharge.

During specialized ophthalmic examination, intense bilateral conjunctival hyperemia was observed, with a granulomatous appearance, presence of lymphoid follicles, chemosis, and thick mucopurulent discharge (Figure 1). On systemic examination, the patient presented with malnutrition, but no cutaneous lesions consistent with sporotrichosis were detected.

Due to the simultaneous and prolonged use of several antibacterial eye drops, it was decided to temporarily suspend topical treatment in order to avoid interference in the collection and laboratory results. A 5-day pharmacological washout period was instituted before performing additional tests, which included a complete blood count, serology for feline immunodeficiency virus (FIV) and feline leukemia virus (FeLV), bacterial culture and conjunctival cytological scraping.

The main clinical hypotheses considered were feline herpesvirus type 1 (FHV-1), fungal infection and ocular bacterial resistance. The tests for FIV and FeLV were negative, and the complete blood count showed no significant changes. The conjunctival scraping revealed the presence of inflammatory cells, such as polymorphonuclear leukocytes and macrophages, but without identification of fungal structures.

As an empirical approach, treatment was started with acyclovir ophthalmic ointment 50 mg/g, administered bilaterally every 6 h for 5 days. After this period, the antibiogram of the microbiological culture of the conjunctiva identified *Enterococcus* sp. with exclusive sensitivity to gentamicin (10 mcg). Based on this finding, gentamicin sulfate eye drops were instituted, applied every 6 h for 15 days.

**Figure 2:**
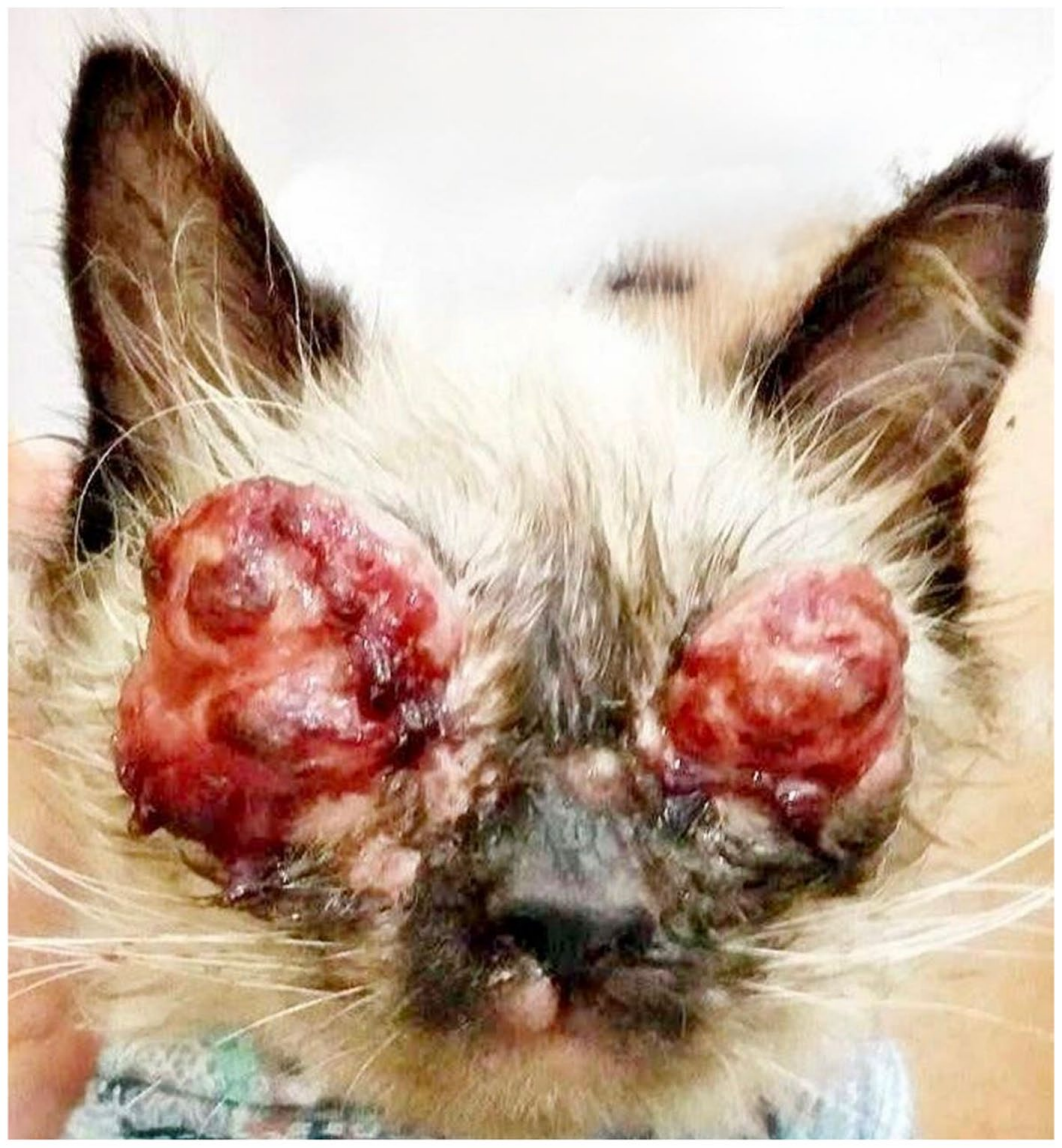
Patient presentation 15 days after the start of interventions, showing significant worsening. Ulcerated, proliferative, and nodular lesions with a granulomatous appearance are observed bilaterally on the upper and lower eyelids, with areas of necrosis, hemorrhagic crusts, and intense serosanguinous exudate, resulting in eyelid deformity and obliteration of the palpebral fissure. Cutaneous lesions are also noted on the upper lip and snout region.

However, the owner, on her own initiative, used eye drops containing gentamicin associated with dexamethasone and, at the same time, administered systemic corticosteroids when she noticed an exacerbation of the ocular inflammation. Fifteen days after starting these medications, she returned to the clinic reporting worsening of the ophthalmic condition, with more extensive and severe lesions (Figure 2). In addition, she observed the emergence of an ulcerative lesion on the left thoracic limb, which prompted an urgent reevaluation.

**Figure 3:**
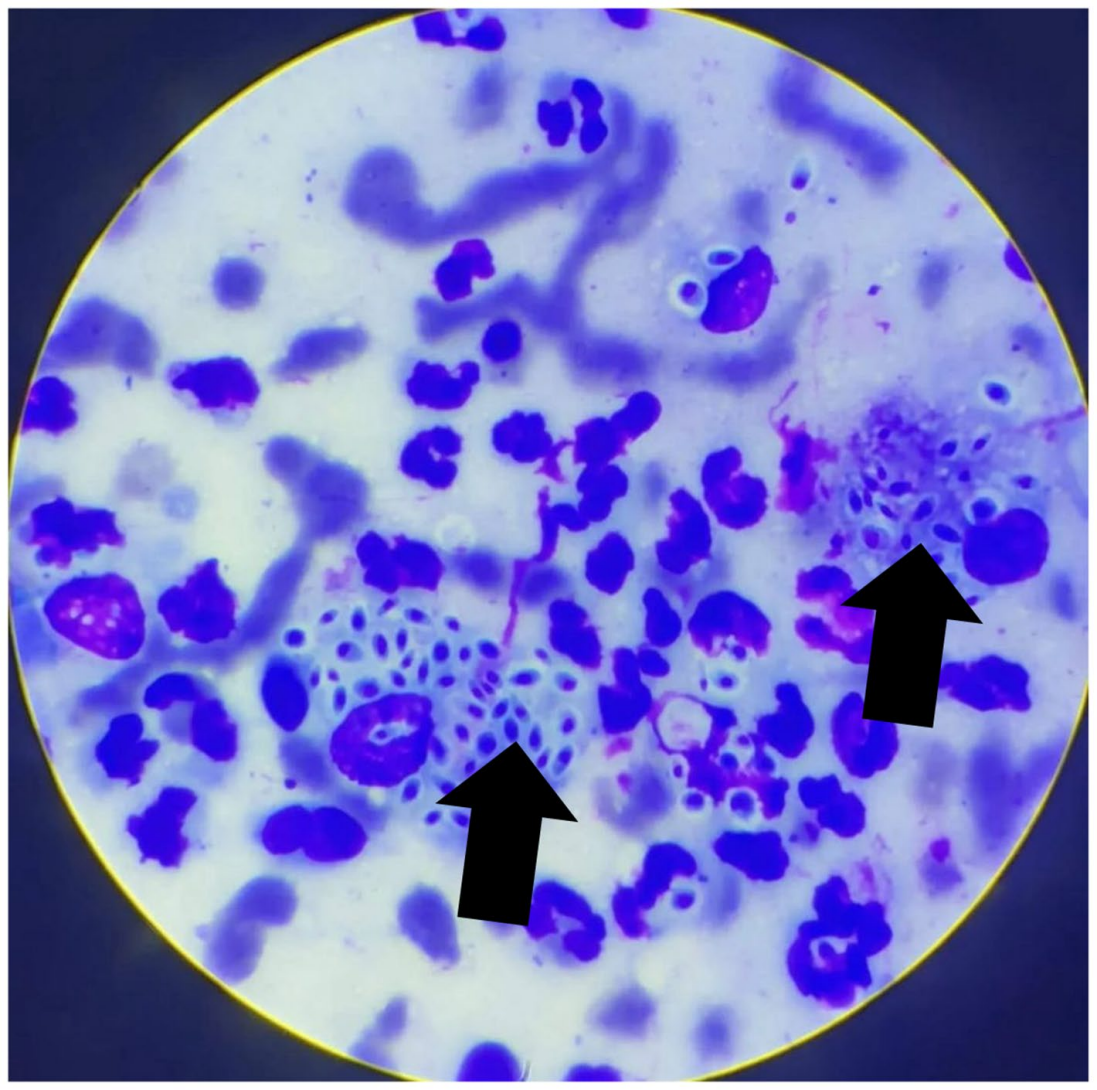
Cytological image from a sample obtained from the ocular conjunctiva by fine-needle aspiration, stained with a rapid Romanowsky-type stain. A pyogranulomatous inflammatory infiltrate is evident, composed of numerous degenerated neutrophils and macrophages. Rounded, sometimes ovoid to fusiform (“cigar-shaped”) yeast-like structures, surrounded by a thin clear halo and with a basophilic center, compatible with *Sporothrix* spp., are observed (arrows). Tissue architecture is not preserved, consistent with exfoliative material. Magnification: 1000 × .

At the follow-up consultation, fine-needle aspiration cytology of the ocular conjunctivae and the cutaneous lesion was performed, revealing ovoid structures consistent with yeasts of the genus *Sporothrix* spp. (Figure 3). For definitive confirmation, conjunctival material was submitted to the microbiology laboratory for fungal culture on Sabouraud dextrose agar (SDA) supplemented with chloramphenicol, incubated at 25 °C under aerobic conditions. After 15 days, growth of dark, wrinkled, filamentous colonies were observed. Microscopic analysis revealed thin, septate hyphae with conidia arranged in characteristic rosette or sympodial patterns. These findings are considered typical of *Sporothrix* spp. and help differentiate it from other dimorphic fungi, such as *Histoplasma* sp. (small, tubercular microconidia) and *Blastomyces* sp. (rounded, thick-walled conidia), which do not exhibit the rosette arrangement. Nevertheless, we recognize that molecular methods would provide more robust confirmation at the species level, although they are often not routinely available in clinical practice.

**Figure 4:**
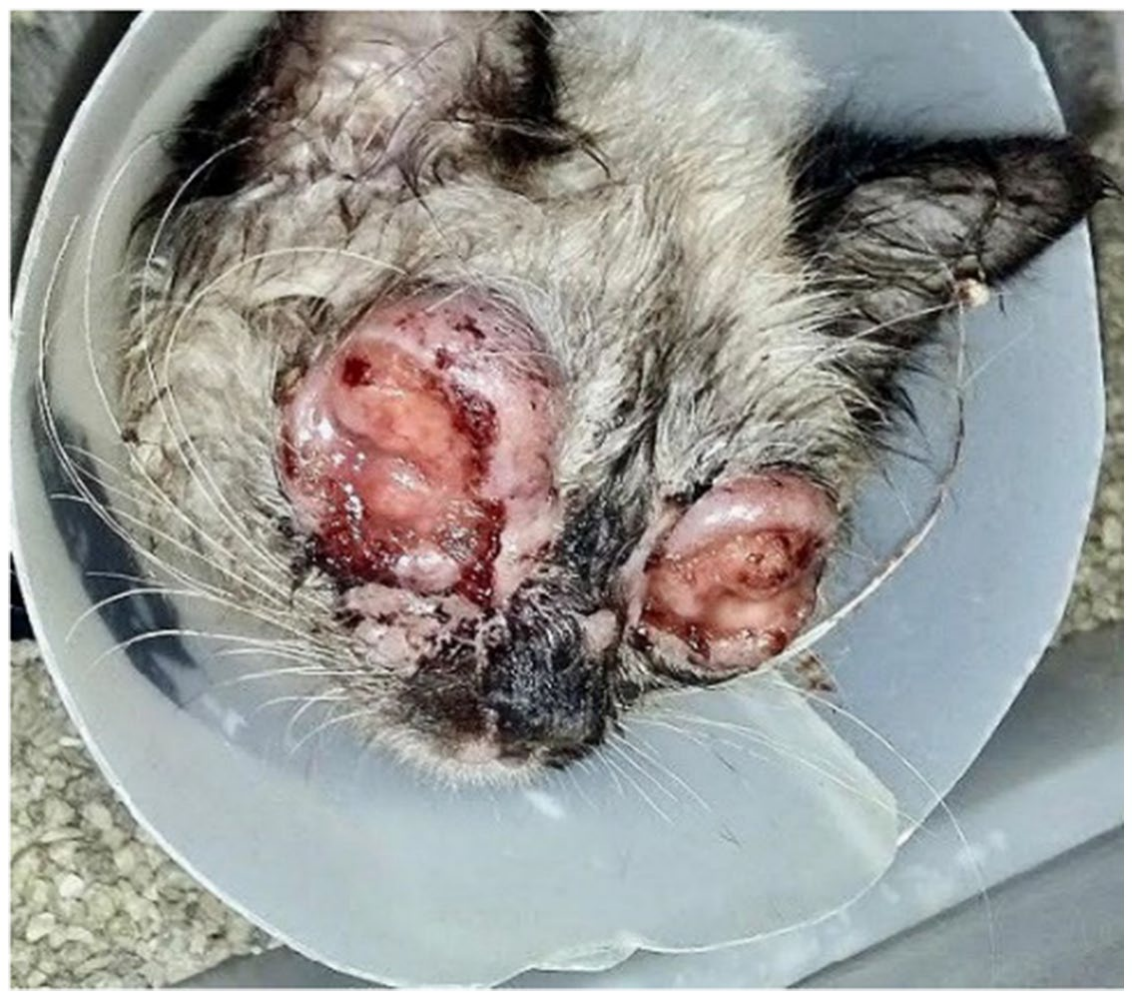
Patient presentation 35 days after the start of antifungal treatment. Exuberant ulcerated and granulomatous lesions persist along the upper and lower eyelid margins. Friable nodular masses with partial necrosis and serosanguinous discharge are observed, although a slight reduction in periocular edema is noted. The clinical picture remains compatible with intense inflammatory activity, with no clinical evidence of re-epithelialization.

A therapeutic protocol with systemic itraconazole (10 mg/kg, PO, SID), associated with S-adenosylmethionine (SAMe) 90 mg/animal, PO, SID, was immediately initiated in order to preserve liver function during prolonged antifungal treatment. After 35 days, the mucopurulent secretion disappeared, but only a slight regression of the conjunctival lesions was observed (Figure 4). The itraconazole dosage was then adjusted to 50 mg/animal, PO, SID.

**Figure 5:**
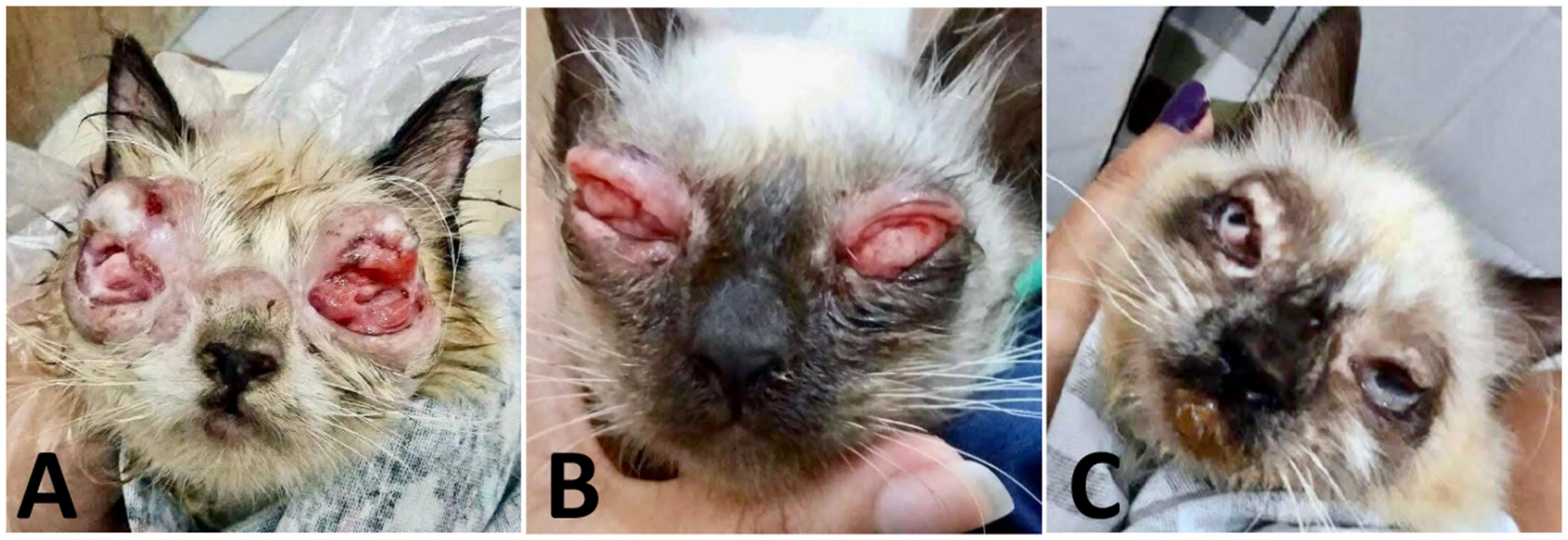
Case evaluation. **(A)** 65 days after the start of treatment: significant reduction in lesion volume and exudate, less friable masses, and onset of re-epithelialization. **(B)** 75 days after the start of treatment: advanced regression of proliferative tissue, with partial epithelialization and decreased friability. Nodular thickening persists. **(C)** 120 days: remission of active lesions, with bilateral symblepharon, eyelid fibrosis, and anatomical deformation consistent with chronic healing.

Thirty days after dose adjustment, the clinical response remained unsatisfactory (Figure 5A). Therefore, a compounded topical lotion of itraconazole 2% associated with beta-glucans (Macrogard®) at 5% was introduced, applied twice a day for 30 days. After 10 days of use, there was significant improvement, with evident regression of the conjunctival alterations (Figure 5B). After 30 days, the lesions were clinically resolved. It was decided to extend the topical and systemic antifungal therapy for another 30 days, totaling 4 months and 5 days of treatment. At the end of the therapy, bilateral symblepharon was observed, for which surgical correction was indicated; and eyelid fibrosis as a consequence of intense conjunctival inflammation (Figure 5C).

## Discussion

Ocular sporotrichosis in felines is a rare and poorly documented condition, especially when it presents as isolated bilateral conjunctivitis in young animals. Most reports describe immunocompromised adult cats with periorbital skin lesions that extend to the conjunctiva (12, 13). In the present case, the exclusively conjunctival and bilateral manifestation in a 2-month-old kitten was configured as an atypical presentation, which initially diverted the clinical suspicion and delayed the mycological diagnosis. Another important point was the initial absence of cutaneous lesions, a situation that underscores the rarity of this presentation. Lesions on the upper lip and facial skin appeared only later. This delayed the diagnostic confirmation, which was initially considered only after clinical progression. In view of this, the possibility of a superficial fungal infection or autoinoculation was initially considered. This type of atypical presentation, although uncommon, has already been briefly discussed in the literature (14).

From an epidemiological perspective, it was not possible to establish a direct chain of transmission. The infection is believed to have occurred before rescue when the animal was living on the streets, possibly through contact with contaminated environmental substrates, since the fungus is saprophytic, present in soil and decomposing organic matter (1, 10). This hypothesis is reinforced by the lack of history of contact with other felines or sick humans in the subsequent household. It is also noteworthy that there is no evidence of transplacental transmission in felines, making environmental exposure the most plausible explanation for the early infection observed (2).

Before the specialist evaluation, the patient had been subjected to concomitant administration of eye drops from different pharmacological classes, such as aminoglycosides, fluoroquinolones, nonsteroidal anti-inflammatory drugs and broad-spectrum antibiotics. This empirical polymedication without adequate guidance can cause pharmacodynamic antagonisms, local adverse effects and masking of clinical signs, making diagnostic evaluation difficult (15, 16). Therefore, it was necessary to establish a pharmacological “wash out” period, with suspension of topical medications before collecting samples for cytology and culture, in order to avoid false-negative results (17).

The first conjunctival cytology revealed only an inflammatory infiltrate, without visible fungal structures—a common finding in the early stages of infection, when the fungal load is low or the yeasts are confined to deep tissue layers (18). Diagnostic confirmation was only possible after clinical progression, with the appearance of an ulcerated skin lesion, and confirmation by fungal culture. The combination of cytology and culture reinforces the importance of complementary methods, especially in atypical cases (12, 19).

The inadvertent use of topical and systemic corticosteroids by the owner significantly worsened the clinical picture, promoting local and systemic immunosuppression, which favored fungal dissemination (18, 20). Paradoxically, this worsening ended up contributing to the morphological evidence of the agent and the definition of the diagnosis. A similar situation is described in the human medical literature, in which corticosteroid-induced immunosuppression intensifies fungal lesions and facilitates the visualization of the pathogen (21).

Due to the patient’s weakened physical condition—a kitten only 2 months old, weighing 300 g and in a state of malnutrition—a conservative dose of itraconazole (10 mg/kg, PO, SID) was initially chosen, with the aim of minimizing hepatic risks and ensuring pharmacological safety. Kittens have hepatic and enzymatic immaturity, which can alter the metabolism of triazole antifungals and increase the risk of toxicity (22). In addition, itraconazole has variable pharmacokinetics in young and weakened felines, and caution is recommended when adjusting the dose, especially in prolonged treatments (23). The choice of the initial dose aimed to balance therapeutic efficacy and tolerability, with continuous clinical monitoring and adjustment according to the observed response. After the first 35 days of treatment, although there was a slight clinical improvement, persistent conjunctival changes and slow regression of the lesions were observed, indicating a suboptimal response to the initial dose.

Considering that, at the time, the patient had already been fully housebroken, had shown nutritional recovery and was receiving liver support with S-adenosylmethionine (SAMe), the dose was adjusted to 50 mg/animal, administered once a day. This titration followed recommendations that indicate this dosage as safe and effective in felines weighing up to 3 kg, especially in refractory or long-term infections (2). The objective was to achieve adequate therapeutic concentrations in ocular and cutaneous tissues without compromising liver function, which remained stable during clinical follow-up. Dosage adjustment also reflected the need for a more aggressive approach to the progression of sporotrichosis, respecting the principles of individualized pharmacotherapy.

The clinical response to systemic itraconazole remained limited until the introduction of topical antifungal therapy associated with beta-glucans (Macrogard®), which resulted in significant improvement in the condition. Beta 1,3/1,6-glucans are immunomodulatory polysaccharides that act by activating innate immunity receptors, such as Dectin-1 and Toll-like, present in macrophages, neutrophils, and dendritic cells, promoting phagocytosis and the production of pro-inflammatory cytokines, in addition to stimulating adaptive immunity (24, 25).

Recent studies (2023–2024) in humans have demonstrated the usefulness of measuring (1,3)-*β* d glucan in ocular fluids—such as tears and vitreous humor—for the diagnosis of fungal keratitis and endophthalmitis, with sensitivity between 82% and 100% and specificity greater than 84%, being especially effective in cases of infection by filamentous fungi (26, 27). In addition, cationic derivatives of β glucans have been evaluated as adjuvant antifungal agents in experimental models, showing direct activity against pathogens such as *Fusarium* spp. and *Candida albicans*, with low toxicity (28).

In veterinary medicine, beta-glucans have been used as safe and effective adjuvants to enhance antimicrobial treatments, including in fungal eye diseases, promoting clinical improvement, and reducing therapeutic time (29). The combination of systemic therapy with topical application allows a synergistic local and systemic effect, promoting elimination of the pathogen and modulation of the inflammatory response, reducing the risk of sequelae resulting from the exacerbated immune reaction (30).

Despite clinical remission, the patient developed bilateral symblepharon as a sequela of intense conjunctival inflammation, which reinforces the importance of early and targeted interventions to avoid permanent ocular damage.

## Conclusion

This report describes a rare case of ocular sporotrichosis in a feline, presenting exclusively with bilateral conjunctival involvement in a young kitten. Initial management with multiple topical medications and the inadvertent use of corticosteroids worsened the condition and delayed definitive diagnosis. Confirmation of the causative agent through fungal culture, in combination with cytology, was crucial for diagnosis. Combined treatment with oral itraconazole, topical antifungal therapy, and *β*-glucan immunomodulation resulted in clinical remission after 4 months. This case highlights the importance of early etiological identification, rational use of antifungals, and individualized follow-up—not only to ensure clinical resolution but also to minimize complications and prevent sequelae such as symblepharon and eyelid fibrosis. Furthermore, this report serves as a warning to clinicians in endemic areas, emphasizing that sporotrichosis should be considered as a differential diagnosis in cases of feline conjunctivitis.

## Data Availability

The original contributions presented in the study are included in the article/supplementary material, further inquiries can be directed to the corresponding author.
